# The complete mitochondrial genome of a parasite at the animal-fungal boundary

**DOI:** 10.1186/s13071-020-3926-5

**Published:** 2020-02-17

**Authors:** Salma Sana, Emilie A. Hardouin, Richard Paley, Tiantian Zhang, Demetra Andreou

**Affiliations:** 1 Department of Life and Environmental Sciences, Faculty of Science and Technology, Bournemouth University, Talbot Campus, Poole, BH12 5BB UK; 2 Division of Crop Plant Genetics, University of Göttingen, 37075 Göttingen, Germany; 3 Centre for Environment Fisheries and Aquaculture Science, Barrack Road, Weymouth, DT4 8UB UK

**Keywords:** Mitochondrial DNA, Mesomycetozoea, Parasite, *Sphaerothecum destruens*, Topmouth gudgeon, Invasive, Animal-fungal boundary, Dermocystida, *Pseudorasbora parva*

## Abstract

**Background:**

*Sphaerothecum destruens* is an obligate intracellular fish parasite which has been identified as a serious threat to freshwater fishes. Taxonomically, *S. destruens* belongs to the order Dermocystida within the class Ichthyosporea (formerly referred to as Mesomycetozoea), which sits at the animal-fungal boundary. Mitochondrial DNA (mtDNA) sequences can be valuable genetic markers for species detection and are increasingly used in environmental DNA (eDNA) based species detection. Furthermore, mtDNA sequences can be used in epidemiological studies by informing detection, strain identification and geographical spread.

**Methods:**

We amplified the entire mitochondrial (mt) genome of *S. destruens* in two overlapping long fragments using primers designed based on the *cox*1, *cob* and *nad*5 partial sequences. The mt-genome architecture of *S. destruens* was then compared to close relatives to gain insights into its evolution.

**Results:**

The complete mt-genome of *Sphaerothecum destruens* is 23,939 bp in length and consists of 47 genes including 21 protein-coding genes, 2 rRNA, 22 tRNA and two unidentified open reading frames. The mitochondrial genome of *S. destruens* is intronless and compact with a few intergenic regions and includes genes that are often missing from animal and fungal mt-genomes, such as, the four ribosomal proteins (small subunit *rps13* and *14*; large subunit *rpl2* and *16*), *tatC* (twin-arginine translocase component C), and *ccmC* and *ccmF* (cytochrome *c* maturation protein *ccmC* and heme lyase).

**Conclusions:**

We present the first mt-genome of *S. destruens* which also represents the first mt-genome for the order Dermocystida. The availability of the mt-genome can assist the detection of *S. destruens* and closely related parasites in eukaryotic diversity surveys using eDNA and assist epidemiological studies by improving molecular detection and tracking the parasite’s spread. Furthermore, as the only representative of the order Dermocystida, its mt-genome can be used in the study of mitochondrial evolution of the unicellular relatives of animals.
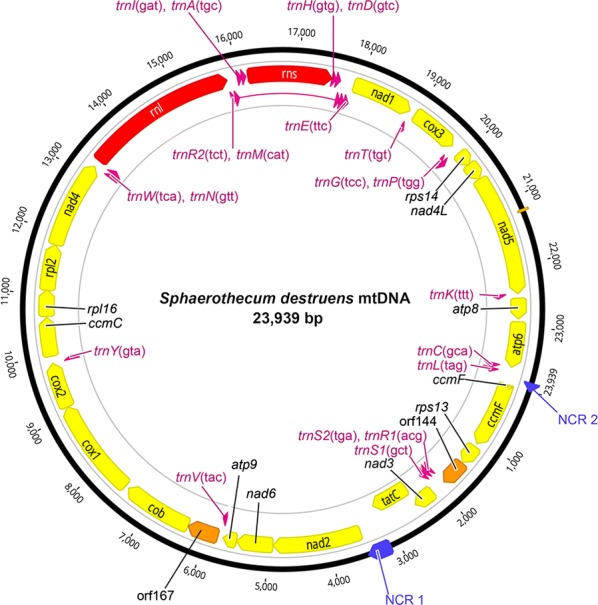

## Background

Introduced parasites can cause significant population declines in susceptible species and generalist parasites in particular, are more likely to be introduced, established and expand their host range [1, 2]. The eukaryotic parasite *Sphaerothecum destruens* is considered a true generalist [1] that can infect and cause high mortalities in freshwater fish species; including commercially important species such as carp and Atlantic salmon [3, 4]. *Sphaerothecum destruens* has been recorded in North America [5–7], Europe [8–12] and China [10]. Sana et al. [10] provided data to support that *S. destruens* was introduced to Europe from China along with the accidental introduction of the invasive fish, topmouth gudgeon *Pseudorasbora parva*. Gozlan et al. [9] has identified *P. parva* as a reservoir host for *S. destruens*, i.e. the parasite can be maintained in *P. parva* and can be transmitted to other fish species whilst not causing disease and mortality in *P. parva*. Since its introduction to Europe, *P. parva* has spread to at least 32 countries from its native range in China [13] and *S. destruens* has been detected in at least 5 introduced *P. parva* populations [8, 10, 12, 14].

*Sphaerothecum destruens* is an asexually reproducing intracellular parasite with a direct life-cycle which involves the release of infective spores to the environment through urine and seminal fluids [15]. The spores can survive and release free-living zoospores in the environment at temperatures ranging from 4 °C to 30 °C [16]. The ability for environmental persistence and its generalist nature, places this parasite as a potential risk to fish biodiversity [17]. Thus, efficient detection of this parasite is essential. Molecular detection using the *18S* rRNA gene is currently the most efficient detection method compared to traditional histology [18]. However, due to the thickened cell wall of *S. destruens*, molecular detection in hosts with low parasite numbers can be difficult [15]. Developing more molecular markers such as mitochondrial DNA markers could improve detection, as there are multiple copies of mitochondria per cell (but note that there are also multiple copies of *18S* rRNA genes per cell as well). Furthermore, mitochondrial genes are increasingly used for environmental DNA (eDNA)-based metabarcoding detection and so sequencing the mt-genome of this fish parasite could increase its detection in eDNA-based metabarcoding studies.

**Fig. 1:**
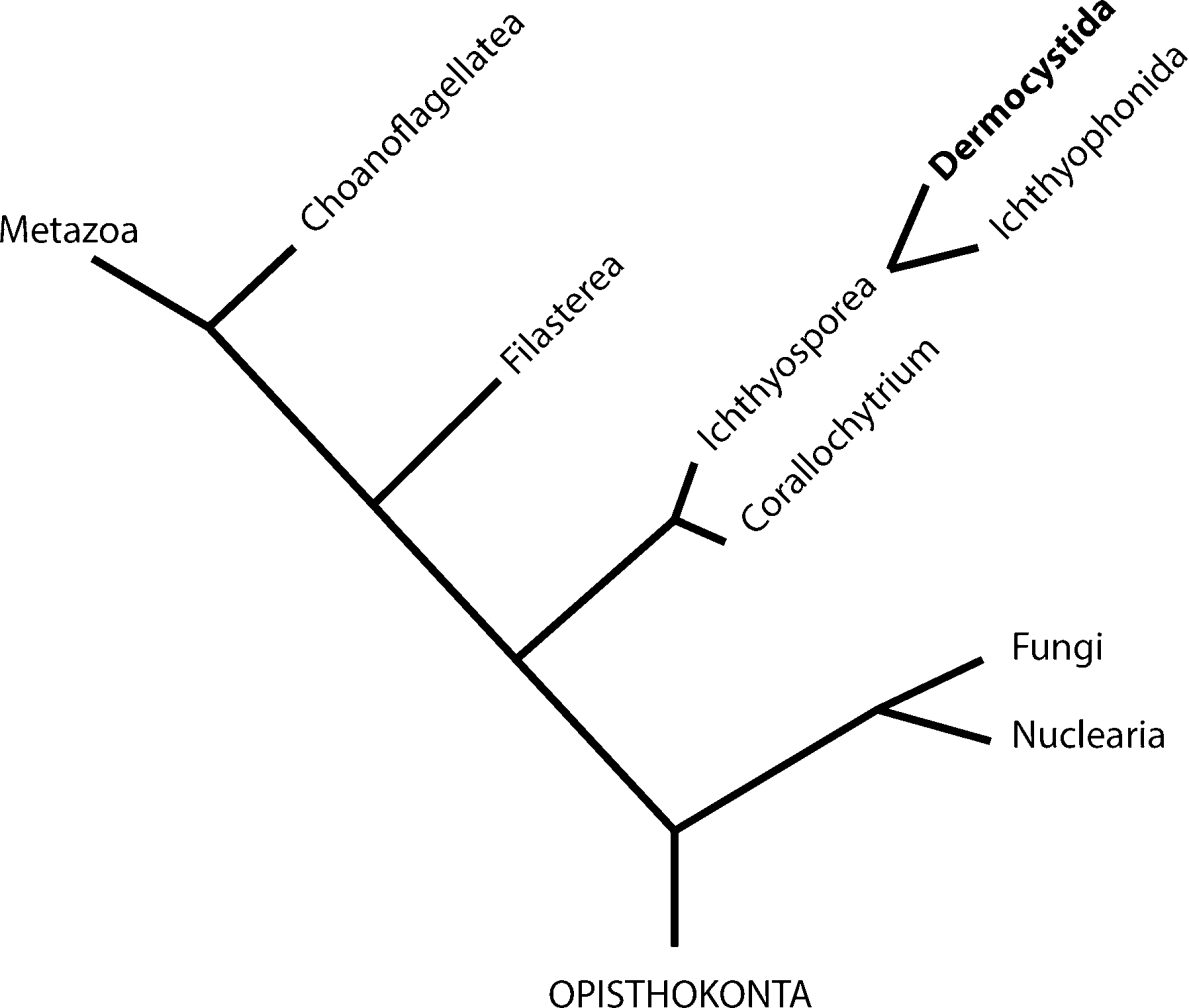
A schematic representation of the phylogenetic position of *Sphaerothecum destruens* (reconstructed from [19, 20]). *Sphaerothecum destruens* belongs to the order Dermocystida which belongs to the class Ichthyosporea. Its taxonomic position is between fungi and animals (Metazoa). Due to the lack of mitochondrial genomes in close relatives, the mitochondrial genome of *S. destruens* was compared to *Amoebidium parasiticum* (Ichthyophonida), *Ministeria vibrans* (Filasterea), *Capsaspora owczarzaki* (Filasterea), *Monosiga brevicollis* (Choanoflagellatea) and *Oscarella carmela* (Demospongiae, Metazoa)

In addition to the importance of *S. destruens* as a potential disease risk for freshwater fishes, its taxonomic position is also evolutionarily important, as it belongs to the class Ichthyosporea (formerly referred to as Mesomycetozoea) which sits at the animal-fungal boundary (Fig. 1) [19]. The class Ichthyosporea consists of two orders, Dermocystida and Ichthyophonida with *S. destruens* grouping within the former [15, 19]. Phylogenomic studies placed *S. destruens* in a new clade termed as “Teretosporea” comprised of Ichthyosporea and *Corallochytrium limacisporum* [20]. Teretosporea was found to be the earliest-branching lineage in the Holozoa [20] and so can be used to provide clues into the origins of higher organisms and mtDNA evolution. Ichthyosporea are difficult to culture, therefore genetic information is often scarce. For example, mitochondrial DNA sequences are lacking for all members of the order Dermocystida.

Here, we have sequenced and present the first complete mt-genome of a species of the Dermocystida, *S. destruens*, in order to develop new tools for the parasite’s detection and provide insights into the parasite’s genome architecture evolution.

## Methods

### DNA extraction and sequencing of *Sphaerothecum destruens* mitochondrial DNA

The *S. destruens* spores used were obtained from *S. destruens* culture in EPC cells [4]*. Sphaerothecum destruens* reproduces asexually so the cultured spores represent clones of a single organism. The partial *18S* rRNA gene from this culture has also been sequenced confirming that this is a culture of *S. destruens* ([4]; GenBank: MN726743). Total genomic DNA was isolated from *S. destruens* spores using the DNeasy Blood and tissue kit (Qiagen, Hilden, Germany). All the steps were performed per manufacturer’s guidelines and DNA was eluted in 100 µl elution buffer and quantified using the Nanodrop (Thermo Fisher Scientific, Waltham, USA). A number of universal mtDNA primers for Metazoa and degenerate primers specific for cnidarians were used to amplify short gene fragments of *S. destruens* mtDNA. The primer pairs were successful in amplifying the short gene fragments of *cox*1 [21], *cob* [22] and *nad*5 [23] of *S. destruens* mtDNA. The mitochondrial fragments spanning the *cob-cox*1 and *cox*1*-nad*5 were amplified using the primer pairs LR-COB-F (5′-ATG AGG AGG GTT TAG TGT GGA TAA TGC-3′) and LR-COX1-R (5′-GCT CCA GCC AAC AGG TAA GGA TAA TAA C-3′); LR-COX1-R3 (5′-GTT ATT ATC CTT ACC TGT GTT GGC TGG AGC-3′) and LR-NAD5-R1 (5′-CCA TTG CAT CTG GCA ATC AGG TAT GC-3′), respectively, with two long PCR kits; Long range PCR kit (Thermo Fisher Scientific) and LA PCR kit (Takara, Clontech, Kasatsu, Japan). The PCR cycling conditions for the mitochondrial fragments were: *cob-cox*1: 94 °C for 2 min, 10× (94 °C for 20 s, 58 °C for 30 s, 68 °C for 7 min), 25× (94 °C for 20 s, 58 °C for 30 s, 68 °C for 7 min (increment of 5 s/cycle) 68 °C for 10 min; and *cox*1*–nad*5 94 °C for 1 min, 16× (94 °C for 20 s, 60 °C for 20 s, 68 °C for 8 min) 19× (94 °C for 20 s, 60 °C, for 20 s, 68 °C for 8 min) 68 °C for 12 min.

The remaining regions of the mitochondrial genome were amplified with the modified step-out approach [24]. The step-out primer used the primers Step-out3 (5′-AAC AAG CCC ACC AAA ATT TNN NAT A-3′) coupled with the species-specific primers LR-cob-R2 (5′-TCA ACA TGC CCT AAC ATA TTC GGA AC-3′) and LR-nad5-R4 (5′-TGG GGC AAG ATC CTC ATT TGT-3′). The PCR cycling conditions were as follows: 94 °C for 1 min, 1× (94 °C for 20 s, 30 °C for 2 min, 68 °C for 8 min), pause to add species-specific primers, 16× (94 °C for 20 s, 65 °C (decrement of 0.3 °C per cycle) for 20 s, 68 °C for 8 min), 19× (94 °C for 20 s, 60 °C for 20 s, 68 °C for 8 min (increment of 15 s per cycle), 68 °C 12 min. Small DNA fragments of up to 1500 bp were directly sequenced. The long fragments which were 12,986 bp and 7048 bp in length were sequenced by primer walk (Beckman Coulter Genomics, Fullerton, USA).

### Gene annotation

Gene annotation of the mitochondrial genome of *S. destruens* was performed using the automated annotation tool MFannot (http://megasun.bch.umontreal.ca/cgi-bin/mfannot/mfannotInterface.pl), followed by visual inspection. Gene annotation was further checked by examining the amino acid sequences of the genes. Genes were translated using the mold, protozoan, and coelenterate mitochondrial code and the mycoplasma/spiroplasma code and aligned with homologous proteins using Clustal W with default options (Gap open cost: 15 and Gap extend cost: 6.66). The 22 tRNA genes were further scanned and secondary structures were generated with MITOS [25]. The annotation for the *tatC* gene was further checked by predicting its secondary structure and comparing it to the secondary structure of two homologous proteins from *Monosiga brevicollis* and *Oscarella carmela.*

### tRNA phylogenetic analysis

tRNA replication was further investigated through phylogenetic analysis using the identified tRNAs from *S. destruens* and the reported tRNAs from its closest relative *A. parasiticum* (GenBank: AF538045 and AF538046; but note that the two species belong to two different orders). Prior to phylogenetic analysis, all tRNA sequences were modified [24]. Specifically, all tRNA sequences had their anticodon sequence and variable loops deleted and CCA was added to all tRNA sequences in which it was missing. The sequences were then aligned using Muscle in Seaview [25, 26] followed by visual inspection. A neighbour-joining tree was constructed in MegaX [27], using 1000 bootstraps and p-distance to calculate evolutionary distance with pairwise deletion option for a total of 56 sequences (22 from *S. destruens* and 24 from *A. parasiticum* (GenBank: AF538045 and AF538046).

## Results

### Gene content and organization

**Fig. 2:**
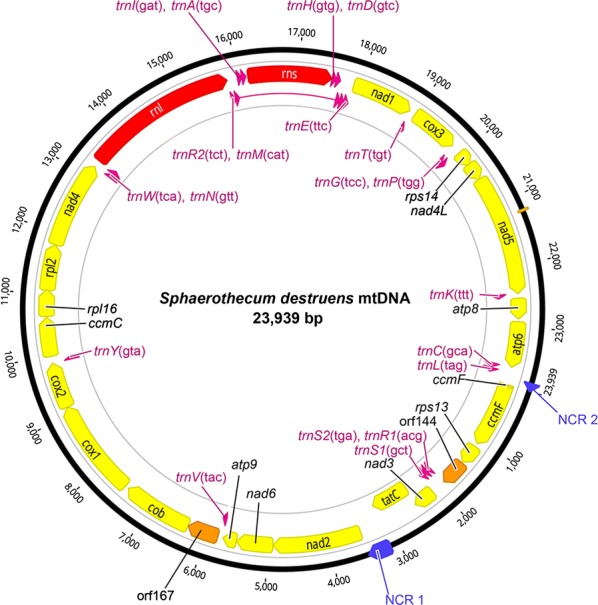
The complete mitochondrial genome for *Sphaerothecum destruens*. All genes are encoded in the same transcriptional orientation. 22 tRNA genes (pink), 2 rRNA genes (red), 19 protein coding genes (yellow), 2 open reading frames (ORFs) (orange)) and 2 non-coding regions (NCR) (blue) are labelled. Twenty-two transfer RNA genes are designated with single-letter amino acid code: A, alanine; C, cysteine; D, aspartic acid; E, glutamic acid; G, glycine; H, histidine; I, isoleucine, K, lysine; L, leucine; M, methionine; N, asparagine; P, proline; R, arginine; S, serine; T, threonine; V, valine; W, tryptophan; Y, tyrosine. Three methionine (M) and two serine (S) and arginine (R) tRNA genes are labelled along with their anticodon sequence

The mitochondrial genome of *S. destruens* was 23,939 bp long with an overall A+T content of 71.2% (Fig. 1). A list of gene order, gene length, and intergenic spacer regions of *S. destruens* mtDNA is given in Table 1. The nucleotide composition of the entire *S. destruens* mtDNA sequences is 40.8% thymine, 31% adenine, 19.7%, guanine and 8.5% cytosine (detailed nucleotide composition is listed in Table 2). It consisted of a total of 47 genes including protein-coding genes (21), rRNA (2) and tRNA (22) and two unidentified open reading frames (ORFs), with all genes encoded by the same strand in the same transcriptional orientation (Fig. 2).

**Table 1:** Mitochondrial genome organization of *S. destruens*

Gene	Position		Size		Codons		Intergenic sequence (bp)
	Start	Finish	No. of nucleotide	No. of amino acid^a^	Initiation	Termination	
*ccmF*	1	1080	1080	359	GTG	TAG	55
*rps*13	1136	1459	324	107	GTG	TAA	3
*orf*144	1463	1897	435				4
*trnS*2	1902	1974	73	–	–	–	1
*trnR*1	1976	2046	71	–	–	–	0
*trnS*1	2047	2126	80	–	–	–	6
*nad*3	2133	2486	354	117	ATG	TAG	− 31
*tatC*	2456	3115	660	219	GTG	TAG	357
*nad*2	3473	4909	1437	478	ATG	TAG	0
*nad*6	4910	5500	591	196	GTG	TAA	13
*atp*9	5514	5738	225	74	ATG	TAA	7
*trnV*	5746	5817	72	–	–	–	3
*orf*167	5821	6324	504				− 1
*cob*	6324	7466	1143	380	ATG	TAG	60
*cox*1	7527	9119	1593	530	ATG	TAA	1
*cox*2	9121	9870	750	249	ATG	TTA	− 1
*trnY*	9870	9944	75	–	–	–	45
*ccmC*	9990	10,622	633	210	ATG	TAA	4
*rpl*16	10,627	11,067	441	146	ATG	TAG	− 11
*rpl*2	11,057	11,806	750	249	TTG	TAA	− 1
*nad4*	11,806	13,236	1431	476	ATG	TAG	0
*trnW*	13,237	13,308	72	–	–	–	2
*trnN*	13,311	13,382	72	–	–	–	− 46
*rrnl*	13,337	15,828	2317	–	–	–	− 4
*trnR*2	15,825	15,897	73	–	–	–	1
*trnM*3	15,899	15,969	71	–	–	–	28
*trnL*	15,998	16,069	72	–	–	–	1
*trnA*	16,071	16,142	72	–	–	–	25
*rrns*	16,168	17,536	1222	–	–	–	− 4
*trnH*	17,533	17,606	74	–	–	–	0
*trnD*	17,607	17,679	73	–	–	–	3
*trnM*2	17,683	17,754	71	–	–	–	0
*trnM*	17,754	17,824	71	–	–	–	1
*trnE*	17,826	17,898	73	–	–	–	6
*nad*1	17,905	18,912	1008	335	TTG	TAG	3
*trnT*	18,916	18,987	72	–	–	–	22
*cox*3	19,010	19,801	792	264	ATG	TAA	2
*trnG*	19,804	19,877	74	–	–	–	7
*trnP*	19,885	19,956	72	–	–	–	1
*rps*14	19,958	20,200	243	80	ATG	TAA	− 7
*nad*4*L*	20,194	20,493	300	99	ATG	TAA	0
*nad*5	20,494	22,458	1965	654	GTG	TAG	− 1
*trnK*	22,458	22,530	73	–	–	–	1
*atp*8	22,532	22,867	336	111	ATG	TAA	45
*atp*6	22,913	23,659	747	248	ATG	TAA	6
*trnC*	23,666	23,738	73	–	–	–	12
*trnL*	23,751	23,822	72	–	–	–	117

^a^Stop codon not included in AA sequence

**Table 2:** Nucleotide composition of mitochondrial genome of *S. destruens*

Nucleotide	Length (bp)	A (%)	C (%)	T (%)	G (%)	A + T (%)	G + C (%)
Entire sequence	23,939	31.0	8.5	40.8	19.7	71.8	28.2
Protein-coding sequences	17,691	28.8	8.0	43.2	20.0	72.0	28.0
rRNA genes sequences	3539	37.9	9.9	33.2	19.0	71.1	28.9
Transfer RNA gene sequences	1601	33.4	11.3	36.2	19.1	69.5	30.5
Non-coding regions	964	38.3	7.3	36.2	18.2	74.5	25.5
NCR 1	357	35.9	11.7	30.8	21.6	66.7	33.3
NCR 2	117	33.3	8.5	35.1	23.1	68.4	31.6

The standard proteins encoded by mitochondria include 13 energy pathway proteins, including subunits 6, 8 and 9 of ATP synthase (*atp*6, *atp*8 and *atp*9), three subunits of cytochrome c oxidase (*cox*1, *cox*2 and *cox*3), apocytochrome b (*cob*) and NADH dehydrogenase subunits 1–6 and 4L (*nad*1, *nad*2, *nad*3, *nad*4, *nad*5, *nad-*6 and *nad*4L). Genes involved in mRNA translation were the small and large subunit rRNAs (*rrns* and *rrnl*). The *S. destruens* mtDNA included genes that are usually absent from standard animal and fungal mtDNAs such as four ribosomal proteins (small subunit *rps*13 and 14; large subunit *rpl*2 and 16), *tatC* (twin-arginine translocase component C), *ccmC* and *ccmF* (cytochrome c maturation protein *ccmC* and heme lyase). The mitochondrial genome of *S. destruens* was intronless and compact with a few intergenic regions. The longest intergenic region was 357 bp and occurred between *tatC* and *nad*2. Several neighbouring genes overlapped by 1–46 nucleotides (Table 1, Fig. 2).

The *tatC* gene (also known as *mttB* and *ymf*16) is present in *M. brevicollis* (Choanoflagellatea) and also reported in only one other animal mt-genome that of *O. carmela* (sponge) (Table 3; [28, 29]). This protein, a component of twin-arginine translocase (tat) pathway, is involved in the transport of fully folded proteins and enzyme complexes across lipid membrane bilayers and is usually present in prokaryotes, chloroplasts and some mitochondria [30]. The *tatC* gene in *S. destruens* is 660 bp long and utilizes GTG as its initiation codon. The derived amino acid sequence of *S. destruens tatC* is most similar to *M. brevicollis tatC* (21%) (Choanoflagellatea) followed by *Reclinomonas americana* (19%) (Jakobid) and *O. carmela* (16%) (Porifera, Metazoa) (Table 4). Secondary structure analysis using TNHMM [31] indicated that the *tatC* gene of *S. destruens* has 6 predicted transmembrane helices at similar locations with the predicted six transmembrane helices for *M. brevicollis* and *O. carmela* (Additional file 1: Figure S1). The *ccmF* protein also known as *yejR* is involved in Heme *c* maturation (protein maturation) and *ccmC* (also known as *yejU*) plays role in heme delivery (protein import).

**Table 3:** Comparison of the mitochondrial genome features of *S. destruens* to other eukaryotes

Taxon	Size (kbp)	Coding portion (%)	No. of tRNAs	Genes coding for						UGA (Trp)	Reference
				rRNAs	Respiratory chain subunits	Ribosomal proteins	Other	No. of ORFs	No. of introns		
*Sphaerothecum destruens* (Dermocystida)	23.9	96.4	22	*rrnl*; *rrns*	*atp*6, 8, 9; *cob*; *cox*1-3; *nad*1-6; *nad*4*l*	*rps*13, 14; *rpl*2, 16	*tatC*; *ccmF*; *ccmC*	2	0	+	This study
*Amoebidium parasiticum* (Icthyophonida)	> 200	~ 20.0	≥ 25	*rrnl*; r*rns*	*atp*6, 8, 9; *cob*; *cox*1-3; *nad*1, 2, 3, 4, 4*L*, 5, 6	*rps*3, 4, 13	–	≥ 24	≥ 21 (I); ≥ 2 (II)	+	[37]
*Ministeria vibrans* (Filasterea)	55.9	80.0	24	*rrnl*; *rrns*	*atp*6,8, 9; *cob*; *cox*1-3*; nad*1-6; *nad*4*l*	*rps*4, 12, 13, 14, 19; *rpl*2, 14, 16, 6, 19	*_*		0	+	[39]
*Capsaspora owczarzaki* (Filasterea)	196.9	28.6	26	*rrnl*; *rrns*	*atp*6, 9; *cob*; *cox*1-3; *nad*1-6; *nad*4*l*	*rps*19, 14, 12; *rpl*2, 14, 16	*ccmF*; *ccmC*	52	1	+	[39]
*Monosiga brevicollis* (Choanoflagellatea)	76.6	46.9	25	*rrnl*; *rrns*	*atp*6, 8, 9; *cob*; *cox*1-*3; nad*1-6; *nad*4*l*	*rps*3, 4, 8, 12–14, 19; *rpl*2, 5, 14, 16	*mttB* (*tatC*)	2	4 (I)	+	[37]
*Oscarella carmela* (Demospongiae; Metazoa)	20.32	~93.7	27	*rrnl*; *rrns*	*atp*6; *cob*; *cox*1-3; *nad*1-6; *nad*4*l*		*tatC*		0		[29]
*Reclinomonas americana* (Jakobida)	65–100	88.0–93.0	25–30	*rrnl*; *rrns*; *rrn*5	*atp*1, 3, 6, 8, 9; *cob*; *cox*1-3; *nad*1-4, 4*L*, *nad*5*-*11; *sdh*2-4	*rps*1*-*4, 7, 8, 10–14, 19; *rpl*1, 2, 5, 6, 10, 11, 14, 16, 18–20, 27, 31, 32, 34, 35	*cox*11, 15; *tufa*; *tat A*, *C*; *ccmA*, *B*, *C*, *F*; *mttB*; *rnpB*; *rpoA*, *D*; *secY*; *rnpB*; *ssrA*; *dpo*	2–22	1(II)	_	[40]

**Table 4:** Comparison of mt protein genes in *Sphaerothecum destruens* (SD) with its close relatives within the Ichthyophonida *Amoebidium parasiticum* (AP), the choanoflagellate *Monosiga brevicollis* (MB), and the Filasterea *Capsaspora owczarzaki* (CO) and *Ministeria vibrans* (MV)

Gene	No. of encoded amino acids^a^					% Amino acid identity				Predicted initiation and termination codons in *S. destruens*	
	SD	AP	MB	CO	MV	SD/AP	SD/MB	SD/CO	SD/MV	Initiation codon	Stop codon
*atp*6	248	249	252	258	247	44	40	41	35	ATG	TAA
*atp*8	111	81	99	-	206	13	20	–	22	ATG	TAA
*atp*9	74	74	73	74	74	70	60	68	65	ATG	TAA
*cox*1	530	507	534	565	529	62	69	64	63	ATG	TAA
*cox*2	249	253	256	251	251	53	55	48	49	ATG	TAG
*cox*3	263	262	263	264	261	56	57	59	46	ATG	TAA
*cob*	380	385	380	381	394	59	62	63	56	ATG	TAG
*nad*1	335	–	343	331	336	–	57	61	52	TTG	TAG
*nad*2	478	–	546	477	451	–	25	17	24	ATG	TAG
*nad*3	117	122	118	117	118	44	55	52	42	ATG	TAG
*nad*4	476	–	498	477	494	–	49	49	41	ATG	TAG
*nad*4*L*	99	99	99	99	109	54	56	54	40	ATG	TAA
*nad*5	654	668	688	638	638	50	48	51	43	GTG	TAG
*nad*6	196	–	228	198	198	–	32	35	30	GTG	TAA
*tatC*	219	–	234	–	–	–	21	–	–	GTG	TAG

^a^Data for *A. parasiticum* and *M. brevicollis* from [28]; data for *C. owczarzaki* and *M. vibrans* from [32]

### Codon usage

Among 21 protein coding genes, 14 genes (*atp*6, *atp*8, *atp*9, *cob*, *cox*1, *cox*2, *cox*3, *nad*2, *nad*3 *nad*4, *nad*4*l*, *rps*14, *rpl*16 and *ccmC*) were inferred to use ATG as initiation codon, 5 genes (*nad*5, *nad*6, *ccmF*, *tatC* and *rps*13) used GTG as a start codon and the remaining *rpl*2 was initiated with TTG. Ten proteins were terminated with the stop codon TAA (*atp*6, *atp*8, *atp*9, *cox*1, *cox*2, *cox*3, *nad*6, *ccmC*, *rps*13, *rps*14), and nine genes used the stop codon TAG (*nad*1, *nad*2, *nad*3, *nad*4, *nad*5, *cob*, *tatC*, *ccmF* and *rpl*16).

### Ribosomal RNA and transfer RNA genes

Genes for the small and large subunits for mitochondrial rRNAs (*rrnS* and *rrnL*, respectively) were present. They were separated by four tRNA genes (*trnA*, *trnI*, *trnM* and *trnR*2). The *rrn*s and *rrnl* (1369 and 2449 bp) had sizes approximately similar to those in *M. brevicollis* (1596 and 2878 bp) and *A. parasiticum* (1385 and 3053 bp). These sizes were comparable to their eubacterial homologs (1542 and 2904 bp in *Escherichia coli*).

**Fig. 3:**
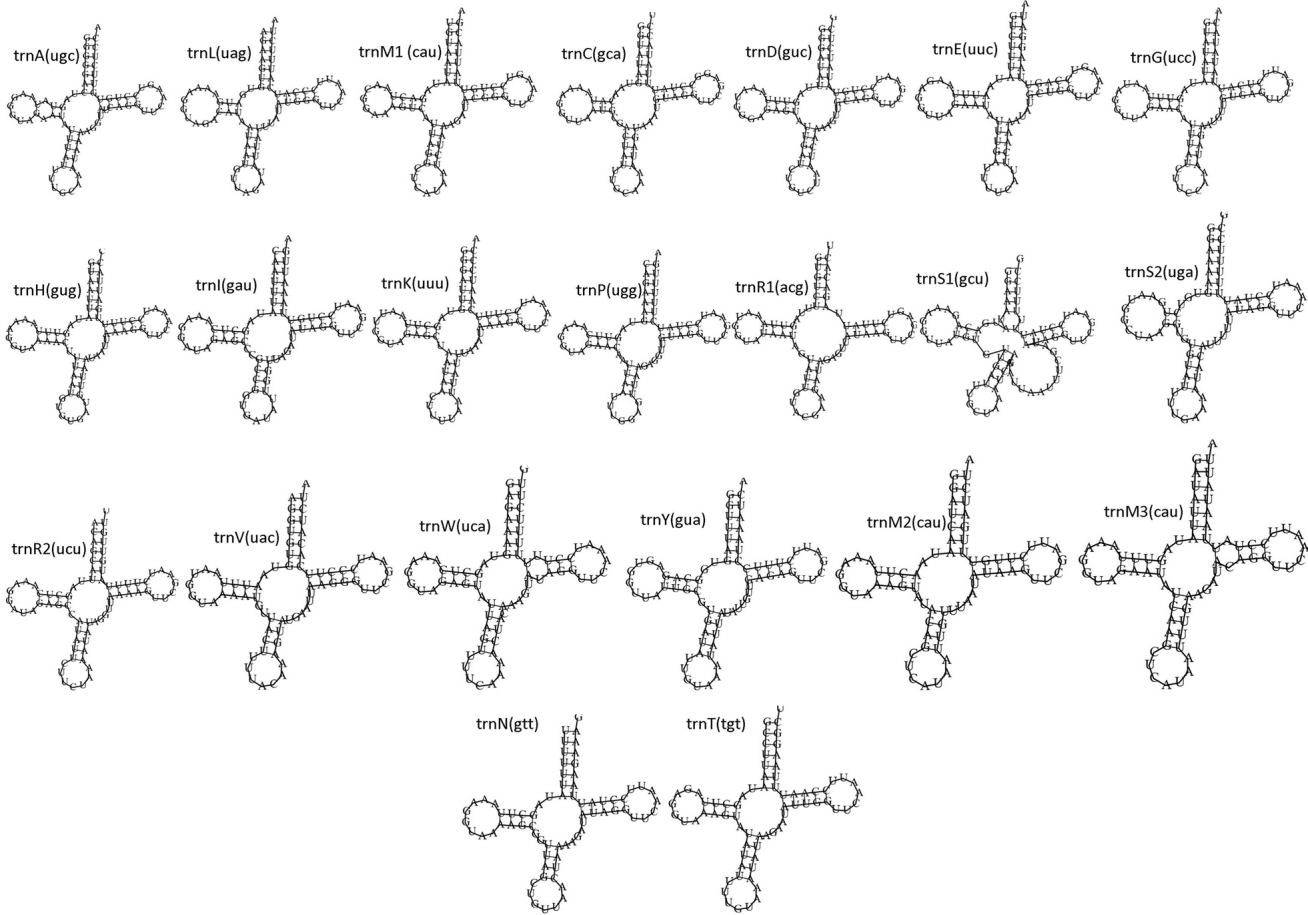
The predicted secondary structures of 22 tRNAs of *Sphaerothecum destruens* mitochondrial DNA generated in MITOS [25] The tRNA stands for *trnA* (transfer RNA alanine), *trnL* (transfer RNA leucine), *trnM*1*-*3 (transfer RNA methionine), *trnC* (transfer RNA cysteine), *trnD* (transfer RNA aspartic acid), *trnE* (transfer RNA glutamic acid), *trnG* (transfer RNA glycine), *trnH* (transfer RNA histidine), *trnI* (transfer RNA isoleucine), *trnK* (transfer RNA lysine), *trnP* (transfer RNA proline), *trnR*1-2 (transfer RNA arginine), *trnS*1*-*2 (transfer RNA serine), *trnV* (transfer RNA valine), *trnW* (transfer RNA tryptophan), *trnY* (transfer RNA tyrosine), *trnN* (transfer RNA asparagine) and *trnT* (transfer RNA threonine)

Twenty-two tRNA genes, including three copies of *trnM*, were identified in *S. destruens* mtDNA. The tRNA genes had a length range of 71–80 bp and their predicted secondary structures had a clover leaf shape (Fig. 3). Three copies of *trnM* (methionine, CAT) had the same length (71 bp) and had the same anticodon - CAT. *trnM*1 was at 1713 bp from *trnM*2, whereas *trnM*2 and *trnM*3 were adjacent (Fig. 2). Two serine and two arginine tRNA genes were differentiated by their anticodon sequence *trnS*1 (GCT) and *trnS*2 (TGA), which were 70% similar, and *trnR*1 (ACG) and *trnR*2 (TCT) which were 63% similar. All the tRNA secondary structures had a dihydrouridine (DHU) arm, a pseudouridine (TΨC) arm and an anticodon stem, except for *trnS*1 (GCT) that had an additional short variable loop. The TΨC and D-loop was comprised of 7 and 7–10 nucleotides, respectively (Fig 3).

### Non-coding regions

The total length of the non-coding regions was 842 bp and was comprised of 32 intergenic sequences ranging in size from 1 to 357 bp. Only two intergenic regions had lengths greater than 100 bp: (i) the non-coding region 1 (NCR 1) was 357 bp long and was located between the *tatC* and *nad2* genes; and (ii) the non-coding region 2 (NCR 2) was 117 bp and was located between the *trnL* and *ccmF* genes (Fig. 2).

### tRNA phylogenetic analysis

**Fig. 4:**
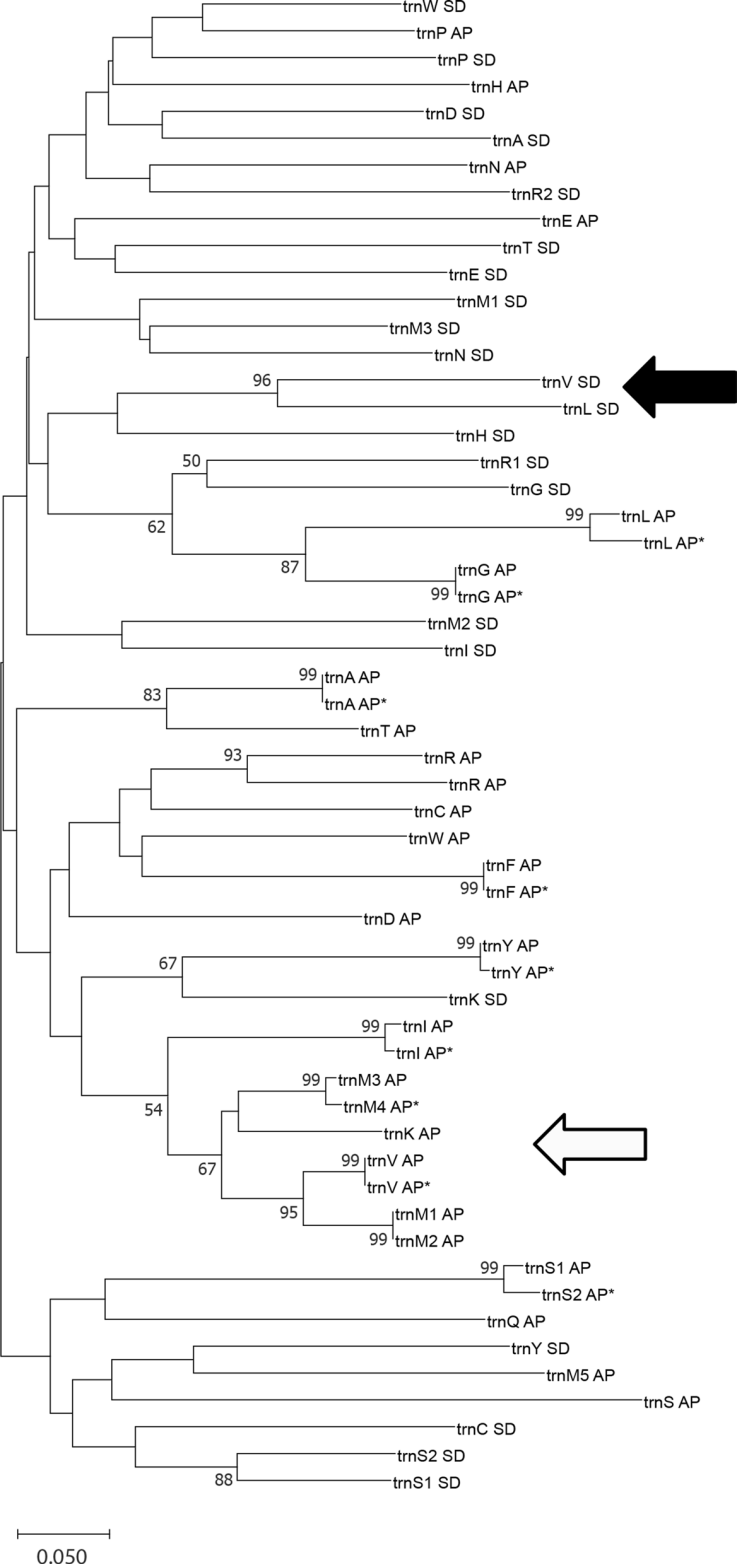
Neighbour-joining treed based on pairwise distances among tRNA genes from *S. phaerothecum destruens* (SD) and *Amoebidium parasiticum* (AP, AF538045; AF*, AF538046) Nucleotides for anticodons and the variable loops were excluded from the analysis. Portions of the tree discussed in the text are indicated by the black and white arrows. Only bootstrap values above 50 are shown

The phylogenetic analysis of the tRNAs of *S. destruens* and *A. parasiticum* showed that the majority of tRNAs grouped by species with few interspecies grouping (Fig. 4). The phylogenetic results suggest that some of the tRNA genes of *S. destruens* could have evolved by gene recruitment; these genes were *trnV* (TAC) and *trnL* (TAG); indicated by the black arrow in Fig. 4. For *A. parasiticum* gene recruitment is suggested for *trnM*, *trnI*, *trnV*, *trnT* and *trnA*, white arrow in Fig. 4, as already suggested by Lavrov & Lang [32].

## Discussion

The mt-genome of *Sphaerothecum destruens* is remarkably compact when compared to other unicellular organisms in similar taxonomic positions and shows the presence of gene overlaps and an absence of both long intergenic regions and repeat sequences. The mt-genome of *S. destruens* has the highest coding portion, 96.4%, among the unicellular relatives of animals, with other members showing much smaller coding regions, e.g. *M. brevicollis* (47%) and *A. parasiticum* (20%). In addition, *S. destruens* had extensive gene loss especially for ribosomal proteins compared to species within the Filasterea and Choanoflagellatea with only four ribosomal genes in its mitochondrial genome and only 22 tRNAs.

The presence of the *tatC* in *S. destruens* represents the first record of this gene within the class Ichthyosporea. *TatC* has also been reported in *M. brevicollis*, a choanoflagellate representing the closest unicellular relatives to multicellular animals, and in multicellular animals such as the sponge *O. carmella* [29]. The *tatC* gene (also known as *ymf*16 and *mttB*) codes for the largest subunit of the twin-arginine transport system pathway and functions in the transport of fully folded proteins and enzyme complexes across membranes [33]. Support for its presence within the *S. destruens* mt-genome was based on sequence similarity and secondary structure comparisons to homologous proteins in *M. brevicollis* and *O. carmela* (Additional file 1: Figure S1). All three homologous *tatC* proteins have a Met initiation codon; with the *tatC* from *S. destruens* and *M. brevicolis* also having the same amino acids following the initiation codon (Ser and Lys). The overall amino sequence similarity between the *tatC* in *S. destruens* and its homologues in *M. brevicollis* and *O. carmella* was 21% and 16%, respectively, and all homologous genes had predicted secondary structures encompassing 6 transmembrane domains consistent with their transmembrane localisation.

Ten genes displayed overlapping regions, with these regions ranging from 1 to 46 nucleotides. Similar levels of gene overlaps have been described in other species [34, 35]. The tRNA *trnN* and *rnl* genes overlap by 46 nucleotides. The overlap is supported by the percentage similarity between the *rnl* sequences of *S. destruens* and *M. brevicollis*, which is 54% (Table 4). The genes *nad*3 and *tatC* overlap by 31 nucleotides and are 44% similar (Table 4). As transcription of the *S. destruens* mitochondrial genome has not been examined, the transcription mechanisms for these proteins can only be hypothesised. A potential mechanism could be the transcription mechanism described for ATPase subunits in mammalian mitochondrial genomes [36].

The closest relative to *S. destruens* which has its mt-genome partially sequenced is *A. parasiticum* which is a member of the order Icthyophonida within the class Ichthyosporea [19]. In contrast to the mt-genome of *S. destruens*, the mt-genome of *A. parasiticum* is large (> 200 kbp) and consists of several hundred linear chromosomes [37]. To date, only 65% of the mt-genome of *A. parasiticum* has been sequenced [37]. In comparison to *A. parasiticum*, the mt-genome of *S. destruens* is at least eight times smaller with all genes encoded by a single circular strand in the same transcriptional orientation. There is a remarkable difference in the coding portion of the genomes between both species with only 20% of the mt-genome of *A. parasiticum* coding for proteins compared to 93% in *S. destruens*. The mt-genome of *S. destruens* contains 47 intron-less genes (including two ORFs) while the mt-genome of *A. parasiticum* intron and gene rich with 44 identified genes and 24 ORFs [37].

Both *S. destruens* and *A. parasiticum* use the mitochondrial UGA (stop) codons to specify tryptophan and have multiple copies of the *trnM* gene. These observed tRNA gene replications are also reported in *M. brevicollis*, *C. owczarzaki* and *M. vibrans* [29, 32, 37]. Similar to *M. brevicollis*, the mitochondrial tRNAs in *S. destruens* did not have a truncated D or T loop structure. The *trnS* of *A. parasiticum* [28], *M. brevicollis* [28] and *S. destruens* does not have a nucleotide at position 8, which connects the aminoacyl and D stems of *trnS*, and in position 26 there is a pyrimidine (uracil) instead of a purine. The *trnS* gene in *S. destruens* also has an adenine instead of uracil in the second nucleotide of its D-loop.

Phylogenetic analysis of the available tRNA sequences of *S. destruens* and *A. parasiticum* suggests that some tRNAs of both species could have evolved by gene recruitment. For *S. destruens* these are *trnV* and *trnL*. Gene recruitment is a process by which a gene is recruited from one isoaccepting group to another changing the tRNA identity [32]. Gene recruitment has been previously reported in *A. parasiticum* for *trnM*, *trnI*, and *trnV* [32]. It is important to note that due to the lack of mitochondrial genomes from close phylogenetic relatives of *S. destruens*, the results of this phylogenetic analysis are limited and must be interpreted with caution. In *S. destruens, trnM*1 and *trnM*3 share a higher nucleotide similarity, 70%, in comparison to *trnM*2 which is 54% and 63%, respectively. The *trnM* replication in *S. destruens* could represent different functions of the methionine tRNAs in protein synthesis and initiation of translation [38]; however, the functional significance remains unknown.

## Conclusions

Mitochondrial DNA sequences can be valuable genetic markers for species detection and are increasingly used in eDNA-based species detection. This is the first record of the mt-genome of *S. destruens*, an important pathogen to freshwater fishes, and the first mt-genome for the order Dermocystida. The availability of this mt-genome should help in the detection of *S. destruens* and closely related parasites in eukaryotic diversity surveys using eDNA. Due to the abundance of mitochondria within cells, mitochondrial DNA could also be used in epidemiological studies by improving molecular detection and tracking the spread of this parasite across the globe [11]. Furthermore, as the only sequenced representative of the order Dermocystida, its mt-genome can be used in the study of the mitochondrial evolution of the unicellular relatives of animals.

## Supplementary information

**Additional file 1: Figure S1.** Secondary structure analysis and comparison of tatC gene of *Sphaerothecum destruens* with *Monosiga brevicollis* and *Oscarella carmela* usingTNHMM [31].

## Data Availability

Data supporting the conclusions of this article are included within the article and its additional file. The generated mitochondrial DNA has been submitted to the GenBank database under the accession number MG832660.

## Abbreviations

ccmC: cytochrome *c* maturation protein
ccmF: cytochrome *c* heme lyase subunit
cob: cytochrome *b*
cox1: cytochrome *c* oxidase subunit 1
eDNA: environmental deoxy ribonucleic acid
mtDNA: mitochondrial DNA
mt-genome: mitochondrial genome
nad5: NADH dehydrogenase subunit 5
NCR: non-coding region
nt: nucleotide
ORF: open reading frame
rRNA: ribosomal ribonucleic acid
rrnl: large subunit ribosomal RNA
rrns: small subunit ribosomal RNA
tatC: twin-arginine translocase component C
trnM: transfer RNA methionine
trnR: transfer RNA arginine
trnS: transfer RNA serine
trnI: transfer RNA isoleucine
trnV: transfer RNA valine
trnT: transfer RNA threonine
trnA: transfer RNA valine

## References

[1] Andreou D, Gozlan RE (2016). Associated disease risk from the introduced generalist pathogen *Sphaerothecum destruens*: management and policy implications. Parasitology..

[2] Fisher MC, Henk DA, Briggs CJ, Brownstein JS, Madoff LC, McCraw SL (2012). Emerging fungal threats to animal, plant and ecosystem health. Nature..

[3] Andreou D, Arkush KD, Guégan JF, Gozlan RE (2012). Introduced pathogens and native freshwater biodiversity: a case study of *Sphaerothecum destruens*. PLoS ONE..

[4] Paley R, Andreou D, Bateman K, Feist S (2012). Isolation and culture of *Sphaerothecum destruens* from Sunbleak (*Leucaspius delineatus*) in the UK and pathogenicity experiments in Atlantic salmon (*Salmo salar*). Parasitology..

[5] Arkush KD, Frasca S, Hedrick RP (1998). Pathology associated with the rosette agent, a systemic protist infecting salmonid fishes. J Aquat Anim Health..

[6] Harrell LW, Elston RA, Scott TM, Wilkinson MT (1986). A significant new systemic disease of net-pen reared chinook salmon (*Oncorhynchus tshawytscha*) brood stock. Aquaculture..

[7] Hedrick R, Friedman C, Modin J (1989). Systemic infection in Atlantic salmon *Salmo salar* with a Dermocystidium-like species. Dis Aquat Organ..

[8] Ercan D, Andreou D, Sana S, Öntaş C, Baba E, Top N (2015). Evidence of threat to European economy and biodiversity following the introduction of an alien pathogen on the fungal-animal boundary. Emerg Microbes Infec..

[9] Gozlan RE, St-Hilaire S, Feist SW, Martin P, Kent ML (2005). Biodiversity: disease threat to European fish. Nature..

[10] Sana S, Williams C, Hardouin EA, Blake A, Davison P, Pegg J (2018). Phylogenetic and environmental DNA insights into emerging aquatic parasites: implications for risk management. Int J Parasitol..

[11] Spikmans F, van Tongeren T, van Alen TA, van der Velde G, den Camp H (2013). High prevalence of the parasite *Sphaerothecum destruens* in the invasive topmouth gudgeon *Pseudorasbora parva* in the Netherlands, a potential threat to native freshwater fish. Aquat Invasions..

[12] Sana S, Hardouin EA, Gozlan RE, Ercan D, Tarkan AS, Zhang T (2017). Origin and invasion of the emerging infectious pathogen *Sphaerothecum destruens*. Emerg Microbes Infec..

[13] Gozlan RE, Andreou D, Asaeda T, Beyer K, Bouhadad R, Burnard D (2010). Pan-continental invasion of Pseudorasbora parva: towards a better understanding of freshwater fish invasions. Fish Fish..

[14] Boitard PM, Charrier A, Labrut S, Jamin M (2017). First detection of *Sphaerothecum destruens* in salmonids in France. Bull Eur Assoc Fish Pat..

[15] Arkush KD, Mendoza L, Adkison M, Hedrick RP (2003). Observations on the life stages of *Sphaerothecum destruens* ng, n. sp., a mesomycetozoean fish pathogen formally referred to as the rosette agent. J Eukaryot Microbiol..

[16] Andreou D, Gozlan R, Paley R (2009). Temperature influence on production and longevity of *Sphaerothecum destruens* zoospores. J Parasitol..

[17] Al-Shorbaji FN, Gozlan RE, Roche B, Robert Britton J, Andreou D (2015). The alternate role of direct and environmental transmission in fungal infectious disease in wildlife: threats for biodiversity conservation. Sci Rep..

[18] Mendonca HL, Arkush KD (2004). Development of PCR-based methods for detection of *Sphaerothecum destruens* in fish tissues. Dis Aquat Organ..

[19] Adl SM, Bass D, Lane CE, Lukes J, Schoch CL, Smirnov A (2019). Revisions to the classification, nomenclature, and diversity of eukaryotes. J Eukaryot Microbiol..

[20] Torruella G, de Mendoza A, Grau-Bové X, Antó M, Chaplin MA, del Campo J (2015). Phylogenomics reveals convergent evolution of lifestyles in close relatives of animals and fungi. Curr Biol..

[21] Folmer O, Black M, Hoeh W, Lutz R, Vrijenhoek R (1994). DNA primers for amplification of mitochondrial cytochrome *c* oxidase subunit 1 from diverse metazoan invertebrates. Mol Mar Biol Biotech..

[22] Boore JL, Brown WM (2000). Mitochondrial genomes of *Galathealinum*, *Helobdella*, and *Platynereis*: sequence and gene arrangement comparisons indicate that Pogonophora is not a phylum and Annelida and Arthropoda are not sister taxa. Mol Biol Evol..

[23] Lavrov DV, Wang X, Kelly M (2008). Reconstructing ordinal relationships in the Demospongiae using mitochondrial genomic data. Mol Phylogenet Evol..

[24] Widmann J, Harris JK, Lozupone C, Wolfson A, Knight R (2010). Stable tRNA-based phylogenies using only 76 nucleotides. RNA..

[25] Edgar RC (2004). MUSCLE: a multiple sequence alignment method with reduced time and space complexity. BMC Bioinform.

[26] Gouy M, Guindon S, Gascuel O (2010). SeaView version 4: a multiplatform graphical user interface for sequence alignment and phylogenetic tree building. Mol Biol Evol..

[27] Kumar S, Stecher G, Li M, Knyaz C, Tamura K (2018). MEGA X: molecular evolutionary genetics analysis across computing platforms. Mol Biol Evol..

[28] Burger G, Lavrov DV, Forget L, Lang BF (2007). Sequencing complete mitochondrial and plastid genomes. Nat Protoc..

[29] Wang X, Lavrov DV (2007). Mitochondrial genome of the homoscleromorph *Oscarella carmela* (Porifera, Demospongiae) reveals unexpected complexity in the common ancestor of sponges and other animals. Mol Biol Evol..

[30] Lee PA, Tullman-Ercek D, Georgiou G (2006). The bacterial twin-arginine translocation pathway. Annu Rev Microbiol..

[31] Krogh A, Larsson B, von Heijne G, Sonnhammer ELL (2001). Predicting transmembrane protein topology with a hidden Markov model: application to complete genomes. J Mol Biol..

[32] Lavrov DV, Lang BF, Wells RD, Bond JS, Klinman J, Masters BSS, Bell E (2016). Mitochondrial genomes in unicellular relatives of animals. Molecular life sciences.

[33] Berks BC, Palmer T, Sargent F (2003). The Tat protein translocation pathway and its role in microbial physiology. Adv Microb Physiol..

[34] Anderson S, Bankier AT, Barrell BG, Debruijn MHL, Coulson AR, Drouin J (1981). Sequence and organization of the human mitochondrial genome. Nature..

[35] Burger G, Plante I, Lonergan KM, Gray MW (1995). The mitochondrial DNA of the amoeboid protozoan *Acanthamoeba castellani* complete sequence, gene content and genome organization. J Mol Biol..

[36] Feagin JE (2000). Mitochondrial genome diversity in parasites. Int J Parasitol..

[37] Burger G, Forget L, Zhu Y, Gray MW, Lang BF (2003). Unique mitochondrial genome architecture in unicellular relatives of animals. Proc Natl Acad Sci USA.

[38] Popova OV, Mikhailov KV, Nikitin MA, Logacheva MD, Penin AA, Muntyan MS (2016). Mitochondrial genomes of Kinorhyncha: trnM duplication and new gene orders within animals. PLoS One..

[39] Lavrov DV, Forget L, Kelly M, Lang BF (2005). Mitochondrial genomes of two demosponges provide insights into an early stage of animal evolution. Mol Biol Evol..

[40] Burger G, Gray MW, Forget L, Lang BF (2013). Strikingly bacteria-like and gene-rich mitochondrial genomes throughout jakobid protists. Genome Biol Evol..

