# Supplementary figures and images for: The complete mitochondrial genome of a parasite at the animal-fungal boundary
Source: Parasit Vectors. 2020 Feb 17;13:81. doi: 10.1186/s13071-020-3926-5 (PMC7027106; doi:10.1186/s13071-020-3926-5)

## Slide 1
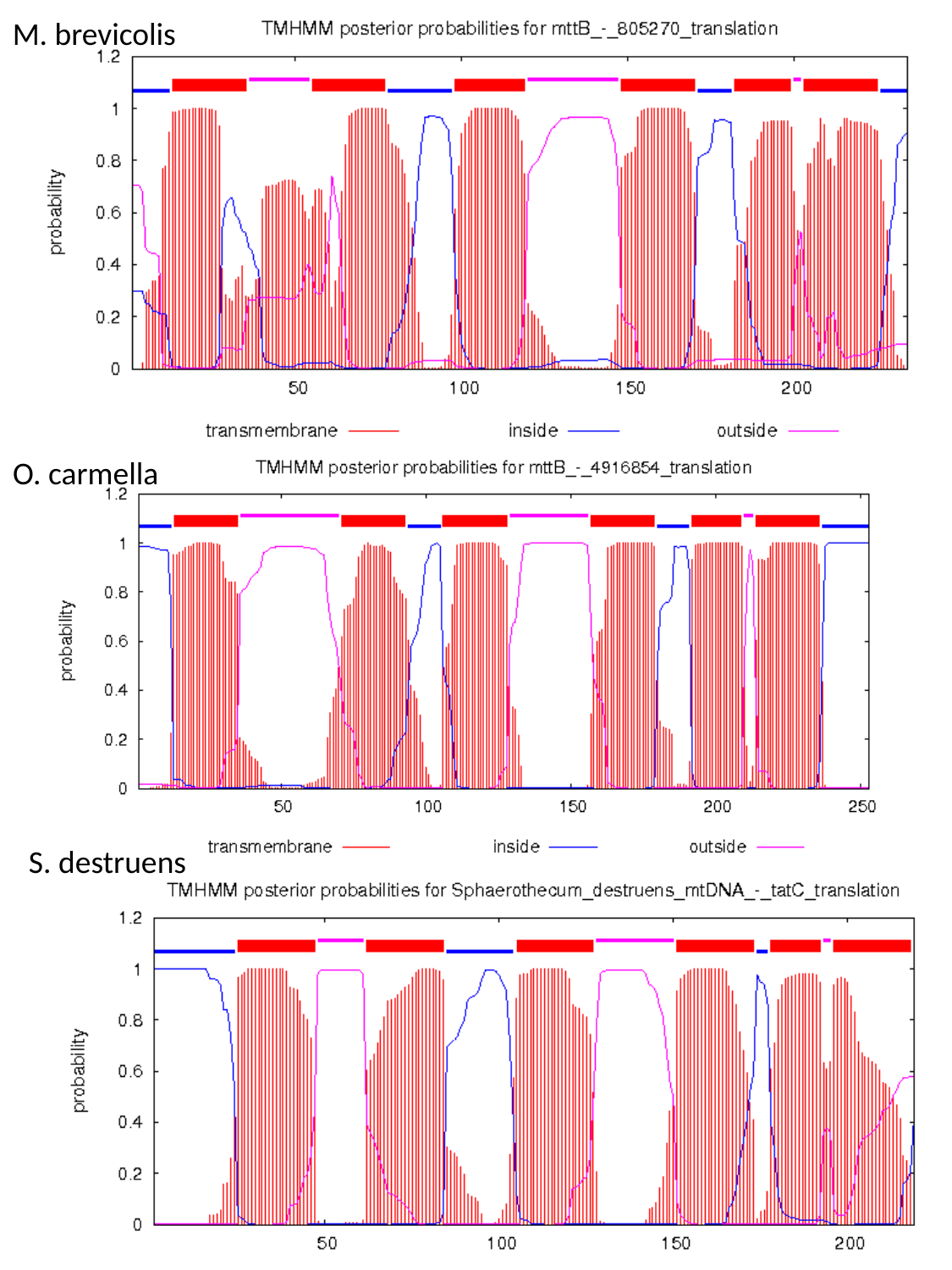

M. brevicolis
O. carmella
S. destruens

Supplement: Supplementary file 1 — Additional file 1: Figure S1. Secondary structure analysis and comparison of tatC gene of Sphaerothecum destruens with Monosiga brevicollis and Oscarella carmela usingTNHMM [31]. [file 13071_2020_3926_MOESM1_ESM.pptx]
